# Changes in the deep vasculature assessed using anterior segment OCT angiography following trabecular meshwork targeted minimally invasive glaucoma surgery

**DOI:** 10.1038/s41598-022-22104-4

**Published:** 2022-10-13

**Authors:** Yoko Okamoto, Tadamichi Akagi, Takanori Kameda, Kenji Suda, Masahiro Miyake, Hanako Ohashi Ikeda, Shogo Numa, Akitaka Tsujikawa

**Affiliations:** 1 Department of Ophthalmology and Visual Sciences, Kyoto University Graduate School of Medicine, Kyoto, Japan; 2 Division of Ophthalmology and Visual Science, Niigata University Graduate School of Medical and Dental Sciences, 1-757, Asahimachi-dori, Chuo-ku, Niigata, 951-8510 Japan

**Keywords:** Eye diseases, Ocular hypertension, Glaucoma

## Abstract

The effect of trabecular meshwork (TM)-targeted minimally invasive glaucoma surgery (MIGS) on the vasculature assessed using anterior segment (AS)-optical coherence tomography angiography (OCTA) has not been established. In this prospective, longitudinal study, we investigated changes in the deep vasculature following TM-targeted MIGS using AS-OCTA for open-angle glaucoma in 31 patients. AS-OCTA images of the sclera and conjunctiva at the nasal corneal limbus were acquired preoperatively and 3 months postoperatively, and the vessel densities (VDs) of the superficial (conjunctival) and deep (intrascleral) layers were calculated. The VDs before and after MIGS were compared, and the factors associated with the change in VD following MIGS were analyzed. The mean deep VD decreased from 11.98 ± 6.80% at baseline to 10.42 ± 5.02% postoperatively (*P* = 0.044), but superficial VD did not change (*P* = 0.73). The multivariate stepwise regression analysis revealed that deep VD reduction was directly associated with IOP reduction (*P* < 0.001) and preoperative IOP (*P* = 0.007) and inversely associated with preoperative deep VD (*P* < 0.001). The deep VD reduction following MIGS was significant in the successful group (21 eyes) (*P* = 0.032) but not in the unsuccessful group (10 eyes) (*P* = 0.49). The deep VDs assessed using AS-OCTA decreased following TM-targeted MIGS, especially in the eyes with good surgical outcomes.

## Introduction

Glaucoma is characterized by progressive degeneration of the retinal ganglion cells^1^. Increased intraocular pressure (IOP) is the main risk factor for glaucoma, and lowering IOP is the only established treatment strategy^2^. Although topical medication is most commonly used for lowering IOP, adverse effects and poor adherence limit its use^3^. Filtering surgeries, including trabeculectomy which is considered the gold standard of glaucoma surgery, are effective for lowering IOP; however, they can lead to sight-threatening complications^4^. To address these limitations, minimally invasive glaucoma surgery (MIGS) was introduced.

Trabecular meshwork (TM)-targeted MIGSs include trabectome surgery^5,6^, microhook ab interno trabeculotomy^7^, suture trabeculotomy^8,9^, and the use of a Kahook dual blade^10^, all of which reduce IOP by relieving the resistance to aqueous humor outflow (AHO) through cleavage or removal of the TM and the inner walls of Schlemm’s canal (SC)^7^. TM-targeted MIGS has advantages such as less invasiveness, faster performance, and significant IOP-lowering effects; however, the variable efficacy of IOP reduction needs to be resolved^11,12^. One of the possible causes for insufficient IOP reduction by TM-targeted MIGS in some cases is thought to be high resistance to AHO after exiting the SC (e.g., high episcleral venous pressure)^12^. Functional assessment of AHO after exiting the SC may be helpful to identify cases in which MIGS is effective.

Imaging of the AHO pathway may facilitate the understanding of the effects of MIGS. Aqueous angiography imaging using indocyanine green, fluorescein, or other dyes has been used to visualize the AHO pathway; however, it has some disadvantages including its relative invasiveness, the need for performance under non-physiological conditions in an operating room, and the difficulty of quantitative evaluation^13–15^. Recently, we reported that anterior segment (AS)-optical coherence tomography angiography (OCTA) can be used to evaluate the scleral and episcleral vasculature associated with AHO^16,17^. Part of the AHO pathway forms a laminar flow with inflowing aqueous humor and red blood cells^1,18^, and AS-OCTA flow signals are derived from flowing red blood cells^19–21^. In a previous study, the deep vasculature, which is mainly composed of episcleral and intrascleral vessels, was assessed with AS-OCTA and was found to be associated with IOP measurements in treated glaucoma patients; this indicates the close relationship between the deep vasculature and IOP in glaucomatous eyes^17^. In addition, our previous report showed that the higher the preoperative AS-OCTA signals in the deep vasculature, the less successful the surgical outcome of MIGS^22^. This indicates that the dense vasculature in the deep layer as observed using AS-OCTA is closely associated with poor function of the aqueous humor. However, the effect of TM-targeted MIGS on the vasculature assessed using AS-OCTA has not been established. Using AS-OCTA, in the present study we investigated the deep vasculature after TM-targeted MIGS and the factors associated with the changes in the deep vasculature.

## Results

Forty-five eyes of 45 consecutive patients were enrolled in the study. Fourteen eyes were excluded from the analysis because of poor-quality AS-OCTA images. Finally, 31 eyes were analyzed in this study (Table 1). All the patients were Japanese, and their mean age was 73.6 ± 9.3 years. Two eyes were normal tension glaucoma. The mean pre-operative IOP was 23.5 ± 7.1 mmHg, with an average of 3.8 glaucoma medications. We observed a reduction in the mean postoperative IOP at 3 months (15.1 ± 4.3 mmHg, *P* < 0.001), 6 months (15.0 ± 4.7 mmHg, *P* < 0.001), and 12 months (15.5 ± 4.2 mmHg, *P* < 0.001).

**Table 1:** Demographic and clinical characteristics of the patients (N = 31).

Age (years)	73.6 ± 9.3 (48–89)
Sex (female/male), no	9/22
Diagnosis (OAG/PPG), no	29/2
Preoperative IOP (mmHg)	23.5 ± 7.1 (15–42)
Axial length (mm)	24.66 ± 1.62 (21.96–29.77)
CCT (μm)	523.3 ± 38.2 (435–600)
VF mean deviation (dB)	− 9.76 ± 6.06 (− 22.33–0.49)
Preoperative glaucoma eye drops, no	3.8 ± 0.9 (2–5)

Data (except for sex and diagnosis) are presented as mean ± standard deviation with the minimum and maximum values in parentheses.

CCT, central corneal thickness; IOP, intraocular pressure; OAG, open-angle glaucoma; PPG, preperimetric glaucoma; VF, visual field.

The preoperative and postoperative vessel densities (VDs) are shown in Table 2. The mean deep VD decreased significantly from 11.98 ± 6.80% at baseline to 10.42 ± 5.02% postoperatively (*P* = 0.044, Wilcoxon signed-rank test), but the superficial VD did not change (baseline, 26.90 ± 7.47%; after surgery, 27.55 ± 7.07%, *P* = 0.73). The power of this comparative analysis was greater than 0.8 for effect sizes higher than a Cohen’s value 0.53.

**Table 2:** Comparison of deep vessel densities before and after minimally invasive glaucoma surgery (N = 31).

Vessel density (%)	Before surgery		3 months after surgery		*P*-value*
	Mean ± SD	95% CI	Mean ± SD	95% CI	
Superficial	26.90 ± 7.47	24.16, 29.64	27.55 ± 7.07	24.95, 30.14	0.73
Deep	11.98 ± 6.80	9.48, 14.47	10.42 ± 5.02	8.59, 12.27	0.044

CI, confidence interval; SD, standard deviation.

*Wilcoxon signed-rank test.

When the factors associated with the change in deep VD were examined, univariate analyses showed that deep VD reduction was significantly associated with greater preoperative superficial VD (*P* = 0.008) and a lower preoperative deep VD (*P* = 0.006) (Table 3). In consideration of small sample size and possible associations among several variables (e.g. preoperative IOP and preoperative deep VD; preoperative IOP and percent change in IOP), the multivariate stepwise regression analysis was performed. The multivariate analysis showed that percent changes in deep VD were directly associated with percent changes in IOP (*P* < 0.001) and preoperative IOP (*P* = 0.007) and inversely associated with preoperative deep VD (*P* < 0.001). The power of this multivariable regression analysis was greater than 0.8 for effect sizes (ʄ^2^) higher than 0.41.

**Table 3:** Factors associated with the percent changes in deep vessel density (VD) (N = 31).

	Univariate analysis			Multivariate analysis*		
	B (95% CI)	β	*P*	B (95% CI)	β	*P*
Percent changes in intraocular pressure (IOP), per 1%	1.230 (− 0.305, 2.765)	0.291	0.11	2.839 (1.320, 4.358)	0.672	** < 0.001**
Preoperative superficial VD, per 1%	0.060 (0.017, 0.104)	0.465	**0.008**			NS
Preoperative deep VD, per 1%	− 0.069 (− 0.116, − 0.021)	− 0.482	**0.006**	− 0.092 (− 0.134, − 0.050)	− 0.647	** < 0.001**
Combined with cataract surgery (vs. single)	− 0.122 (− 0.483, 0.240)	− 0.127	0.50			NS
Preoperative IOP, per 1 mmHg	0.000 (− 0.052, 0.052)	0.0002	> 0.99	0.070 (0.021, 0.120)	0.515	**0.007**
Preoperative anti-glaucoma eye drops, no	0.077 (− 0.339, 0.493)	0.070	0.71			NS
Axial length, per 1 mm	0.096 (− 0.129, 0.321)	0.160	0.39			NS
Central corneal thickness, per 1 μm	− 0.008 (− 0.017, 0.002)	− 0.294	0.11			NS
Visual field mean deviation, per 1 dB	− 0.028 (− 0.088, 0.032)	− 0.173	0.35			NS

Percent changes in IOP = (postoperative IOP 6 months after the surgery − preoperative IOP)/preoperative IOP × 100.

Percent changes in deep VD = (postoperative deep VD − preoperative deep VD)/preoperative deep VD × 100.

B, unstandardized regression coefficient; β, standardized regression coefficient; CI, confidence interval; IOP, intraocular pressure; NS, not significant; VD, vessel density.

*Multiple stepwise regression analysis.

Significant values are in bold.

The patients were classified into two groups based on the postoperative outcome: successful and unsuccessful groups. Twenty-one eyes were allocated to the successful group, and 10 eyes were allocated to the unsuccessful group (Table 4). There were no significant differences in age, sex, axial length, central corneal thickness, preoperative IOP, type of surgical procedure, or combination with cataract surgery in the groups (all *P* > 0.05, unpaired *t*-test and Fisher’s exact test). The difference in surgical procedure and the number of preoperative anti-glaucoma eye drops was marginal (*P* = 0.067 and *P* = 0.063). The number of anti-glaucoma eye drops a year after surgery, the IOP a year after surgery, and the rate of change in the IOP a year after surgery were significantly lower in the successful group than in the unsuccessful group (all *P* < 0.001). In the successful group, the mean preoperative deep VD decreased significantly from 10.20 ± 4.18% (95% confidence interval [CI], 8.29–12.10) at baseline to 8.94 ± 2.76% (95% CI 7.69–10.21) postoperatively (*P* = 0.032). In the unsuccessful group, there was no significant difference in the deep VDs measured pre- and postoperatively (*P* = 0.49). Regarding superficial VD, there were no significant differences between the pre- and postoperative VDs in the successful and unsuccessful groups (all *P* > 0.05). Supplementary Fig. 1 shows the individual deep VDs before and after MIGS for the included cases.

**Table 4:** Comparison of successful and unsuccessful groups (N = 31).

	Successful group (N = 21)	Unsuccessful group (N = 10)	*P*-value
Age at baseline (years)	74.2 ± 9.4	72.1 ± 9.5	0.56
Sex (female/male), no	5/16	4/6	0.42^a^
Axial length (mm)	24.44 ± 1.45	25.11 ± 1.92	0.29
Central corneal thickness (μm)	520.0 ± 33.4	530.1 ± 47.9	0.50
Visual field mean deviation (dB)	− 9.74 ± 6.95	− 9.80 ± 3.90	0.98
Single/combined with cataract surgery, no	10/11	4/6	> 0.99^a^
Surgical procedure (microhook/TOM/s-LOT)	6/3/12	3/5/2	0.067^a^
Preoperative anti-glaucoma eye drops, no	3.6 ± 0.9	4.2 ± 0.6	0.063
Preoperative IOP (mmHg)	24.3 ± 7.5	21.6 ± 6.0	0.33
Anti-glaucoma eye drops 1 year after surgery, no	1.4 ± 1.2	3.4 ± 0.9	** < 0.001**
IOP 1 year after surgery (mmHg)	13.9 ± 3.1	19.6 ± 4.2	** < 0.001**
IOP change rate 1 year after surgery (%)	− 39.8 ± 14.5	− 3.4 ± 23.3	** < 0.001**
Preoperative superficial VD (%)	27.13 ± 7.59	26.44 ± 7.59	0.82
Postoperative superficial VD (%)	27.12 ± 6.72	28.43 ± 8.06	0.64
Preoperative deep VD (%)	10.20 ± 4.18	15.71 ± 9.63	0.11
Postoperative deep VD (%)	8.95 ± 2.76	13.52 ± 7.14	0.078

Data are presented as the mean ± standard deviation unless otherwise indicated.

IOP, intraocular pressure; s-LOT, suture trabeculotomy; TOM, Trabectome; VD, vessel density.

*P*-value: ^a^Calculated using Fisher's exact test; The other values were calculated using an unpaired *t-*test.

Significant values are in bold.

**Figure 1:**
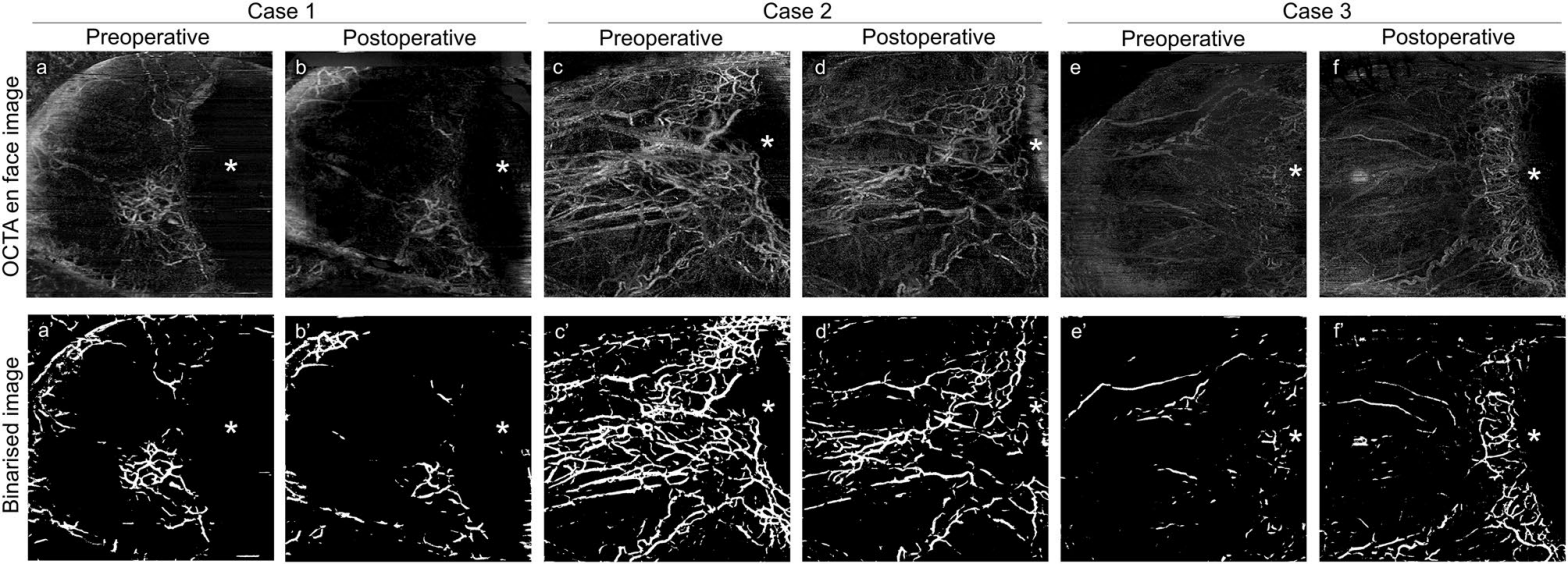
Representative anterior segment (AS)-optical coherence tomography angiography (OCTA) images. (**a**), (**b**) Pre- and postoperative AS-OCTA images of a left eye in the successful group (case 1). The deep vessel density (VD) decreased from 7.32% at baseline to 4.47% postoperatively. (**c**)–(**f**) Mirror-reversed AS-OCTA images of the right eyes in the unsuccessful group (case 2 and case 3). In case 2, the deep VD decreased from 27.50% at baseline to 14.11% postoperatively. In case 3, the deep VD increased from 2.11% to 12.50% postoperatively. (**a′**)–(**f′**) Binarized images of (**a**)–(**f**). Asterisks indicate the location of the cornea.

The AS-OCTA images of the representative cases are shown in Fig. 1. Case 1 was allocated to the successful group, and cases 2 and 3 were allocated to the unsuccessful group. In case 1, the IOP changed from 21 mmHg with three glaucoma medications at baseline to 9 mmHg without glaucoma medication 3 months after surgery, and the deep VD decreased from 7.32% at baseline to 4.47% postoperatively. In case 2, the IOP changed from 33 mmHg with three glaucoma medications at baseline to 21 mmHg with one glaucoma medication 3 months after surgery, and the deep VD decreased from 27.50% at baseline to 14.11% postoperatively. In case 3, the IOP changed from 28 mmHg with four glaucoma medications at baseline to 24 mmHg with four glaucoma medications 3 months after surgery, and the deep VD increased from 2.11 to 12.50% postoperatively. For most of the cases in the successful group, the deep VD decreased after surgery, and no new formation of flow signals was observed postoperatively (Supplementary Fig. 1b and Fig. 1a, b); we observed two different patterns in the unsuccessful group, namely eyes with increased VD and those with decreased VD postoperatively, (Supplementary Fig. 1c, Fig. 1c–f).

## Discussion

This study showed that the density of the deep vasculature assessed using AS-OCTA decreased significantly after TM-targeted MIGS, especially for the eyes that underwent successful MIGS. The reduction of the deep VD was significantly associated with reductions of IOP after MIGS, preoperative deep VD, and preoperative IOP. TM-targeted MIGS is expected to enhance AHO to collector channels; therefore, we postulate that increased AHO in the scleral and episcleral venous plexuses and episcleral veins may lead to the reduction of signal intensity of phase differences detected by AS-OCTA and the consequent reduction of the deep VD.

We previously reported that the AS-OCTA deep vasculature can represent the scleral and episcleral venous plexuses, aqueous veins, and episcleral veins, which were associated with post-TM AHO^16^. The OCTA deep VD was significantly associated with IOP in eyes treated for glaucoma, which indicates that deep VD may be strongly affected by the state of the AHO^17^. The mechanism by which the increase in AHO affects AS-OCTA images is still unknown, but what is important here is that AS-OCTA flow signals are derived from flowing red blood cells and not from AHO^19–21^. Furthermore, in normal eyes, part of AHO forms a laminar flow with inflowing aqueous humor and red blood cells, and pulsatile flow is observed^1,23^. However, in glaucomatous eyes, this mechanism does not function as the disease progresses, and red blood cells reflux from the surrounding blood-containing episcleral tributaries and fill them to the level of the aqueous vein when episcleral vein pressure increases^1,24^. After MIGS, AHO visualized by aqueous humor angiography improves^25^, red blood cells in some episcleral vessels are replaced with aqueous humor, and the concentration of blood cells decreases^18^. In the current study, the deep VD was significantly reduced after MIGS, and this tendency was more significant in the successful group (Supplementary Fig. 1). TM-targeted MIGS reduces IOP by relieving the resistance of the AHO at the TM level, and our results indicate that increased AHO may reduce deep VD by reducing the concentration of red blood cells. The significant association between the changes in the deep VD and IOP following MIGS may be consistent with our speculation. On the other hand, even in the unsuccessful group, the deep VD markedly decreased in some eyes. Although the reason for this inconsistency is not clear, the increased AHO after MIGS might not be enough for surgical success in some eyes, or the other factors (e.g. changes in glaucoma medications) might affect deep VD. Further study may be needed to get a better understanding about the association between OCTA images and AHO function.

Several techniques have been proposed for visualizing the AHO pathway. AS-OCT imaging allows the visualization of AHO structures^26–28^. We previously investigated the changes in AHO structures visualized using AS-OCT after TM-targeted MIGS; however, structural changes in AHO assessed by AS-OCT were not significant after TM-targeted MIGS, and functional assessments of AHO were needed^28^. Fellman et al. reported that an intraoperative episcleral venous fluid wave indicated functional AHO and was significantly associated with the outcome of trabectome surgery, which indicates that changes in AHO may affect the outcome of MIGS^29^. Intracameral injections of some types of dyes, such as trypan blue^15^, indocyanine green^13^, and fluorescein^14,25^, have been used to visualize functional AHO. These methods may facilitate a deeper understanding of the association between AHO and MIGS; however, they have limitations, including their relative invasiveness, the need to be performed in an operating room, and the difficulty associated with quantitative evaluation. AS-OCTA can overcome these limitations; however, the signals derived from flowing red blood cells provided by AS-OCTA should differ from those provided by aqueous angiography, which are derived from AHO.

Some previous studies have focused on flowing red blood cells for evaluating AHO following MIGS, and it has been suggested that aqueous veins with better AHO function are not dilated and filled with red blood cells^18,30,31^. A study using hemoglobin video imaging (HVI) with a modified slit lamp showed that the cross-sectional area of the aqueous column within the episcleral veins can be improved following TM-targeted MIGS^18^. According to the report, the episcleral vein was engorged with blood preoperatively and almost completely disappeared postoperatively owing to aqueous fill and dilution or the displacement of red blood cells. The aqueous fill and dilution or displacement of red blood cells in HVI may result in the reduction of AS-OCTA signals; if this was the case, the result of that study is consistent with ours. We speculate that high deep VD on AS-OCTA reflects vasodilation due to highly concentrated red blood cells and/or high venous pressure, and the low deep VD on AS-OCTA reflects laminar flow due to the good function of AHO. This speculation is consistent with our previous finding that a higher deep VD was significantly associated with a higher IOP in treated glaucomatous eyes^17^. More recently, we reported that preoperative deep VD assessed using AS-OCTA was negatively correlated with the surgical success of TM-targeted MIGS, which indicates that a lower deep VD might be associated with good functioning of post-TM AHO^22^. Future studies using HVI and AS-OCTA will improve our understanding of AS-OCTA.

This study had limitations. First, it was conducted in a single center with a small sample. This small sample size would be not enough for refined analysis. Further validation studies using larger samples are needed to elucidate the association between deep vasculature and TM-targeted MIGS. Second, AS-OCTA images were not compared with aqueous humor angiography or HVI in this study. Further studies are needed to clarify whether our speculation is correct. Given that AS-OCTA is probably affected by red blood cell concentration and that HVI is a non-invasive test, a comparison with HVI would be a possible candidate. Third, AS-OCTA has room for improvement. The currently available AS-OCTA system is designed for the posterior segment, and it requires adjustments in the AS for AS-OCTA imaging. Motion artifacts and the difficulty of matching regions of interest are also major issues concerning AS-OCTA. Future improvement of the equipment and specialized software for the AS may expand the usefulness of AS-OCTA. Fourth, IOP-lowering eye drops may have affected our results. We previously reported that, as a short-term effect, ripasudil instillation increased deep VD as well as superficial VD^32^; therefore, the ripasudil instillation was stopped at least 1 week before surgery and the cessation of ripasudil was maintained after surgery in the current study. Prostaglandin analogues reduce IOP by reducing outflow resistance resulting in increased AHO through the uveoscleral pathway, and β-blockers and carbonic anhydrase inhibitors reduce aqueous humor production^2^. These medications also may affect deep VD. There was no significant difference in the number of anti-glaucoma eye drops between the successful and unsuccessful groups (Table 4), and our previous study showed that the number of glaucoma medications was not significantly associated with deep VD in eyes treated for glaucoma^17^. However, the associations between glaucoma medications and deep VD have not been fully revealed. Further investigations are needed to clarify this.

In conclusion, our study showed that AS-OCTA can detect changes in the deep vasculature following TM-targeted MIGS in sectors where the TM was incised or removed. The deep VD decreased after MIGS, especially for the eyes with a successful postoperative course, and the change in the deep VD was significantly associated with the effect of surgery. Although AS-OCTA still has room for improvement, it is a promising tool for evaluating AHO after MIGS.

## Materials and methods

This study included patients who were enrolled in two ongoing prospective studies at Kyoto University Hospital: the Kyoto University Glaucoma Progression Study (registered with the University Hospital Medical Information Network [UMIN] Clinical Trial Registry of Japan [UMIN000019854]) and the Clarification of Eye Diseases using OCTA (UMIN000028853). This study was approved by the Institutional Review Board and Ethics Committee of the Kyoto University Graduate School of Medicine, and it adhered to the principles of the Declaration of Helsinki. All participants provided written informed consent.

### Participants

We included patients with open-angle glaucoma, preperimetric glaucoma, or ocular hypertension who underwent TM-targeted MIGS at Kyoto University Hospital between October 1, 2017, and January 31, 2020. The inclusion criteria were as follows: open angle on gonioscopy, best-corrected visual acuity of 20/40 or better at baseline, preoperative IOP of 15 mmHg or higher, no history of intraocular surgery other than cataract surgery, and no ocular diseases other than cataract. When both eyes met the inclusion criteria, the eye treated first was included for the analysis. The exclusion criteria were as follows: follow-up for less than 12 months after surgery and poor-quality AS-OCTA images.

The patients had undergone a preoperative comprehensive ophthalmic examination including slit-lamp and gonioscopic examinations, measurement of best-corrected visual acuity (using a 5-m Landolt chart), axial length (IOLMaster 500; Carl Zeiss Meditec, Dublin, California, USA), central corneal thickness (SP-3000; Tomey, Tokyo, Japan), Goldmann applanation tonometry, standard automated perimetry (Humphrey Visual Field Analyzer; Carl Zeiss Meditec) with the 24–2 Swedish interactive threshold algorithm standard program, circumpapillary retinal nerve fiber layer measurements using a Spectralis HRA + OCT scanner (Heidelberg Engineering, Heidelberg, Germany), and AS-OCTA examinations. Clinical information was collected during the follow-up visits at 1, 3, 6, 9, and 12 months postoperatively. For each follow-up visit, the IOP results were obtained using a Goldmann applanation tonometer, and the number of postoperative anti-glaucoma eye drops and any complications were recorded.

### AS-OCTA examination

**Figure 2:**
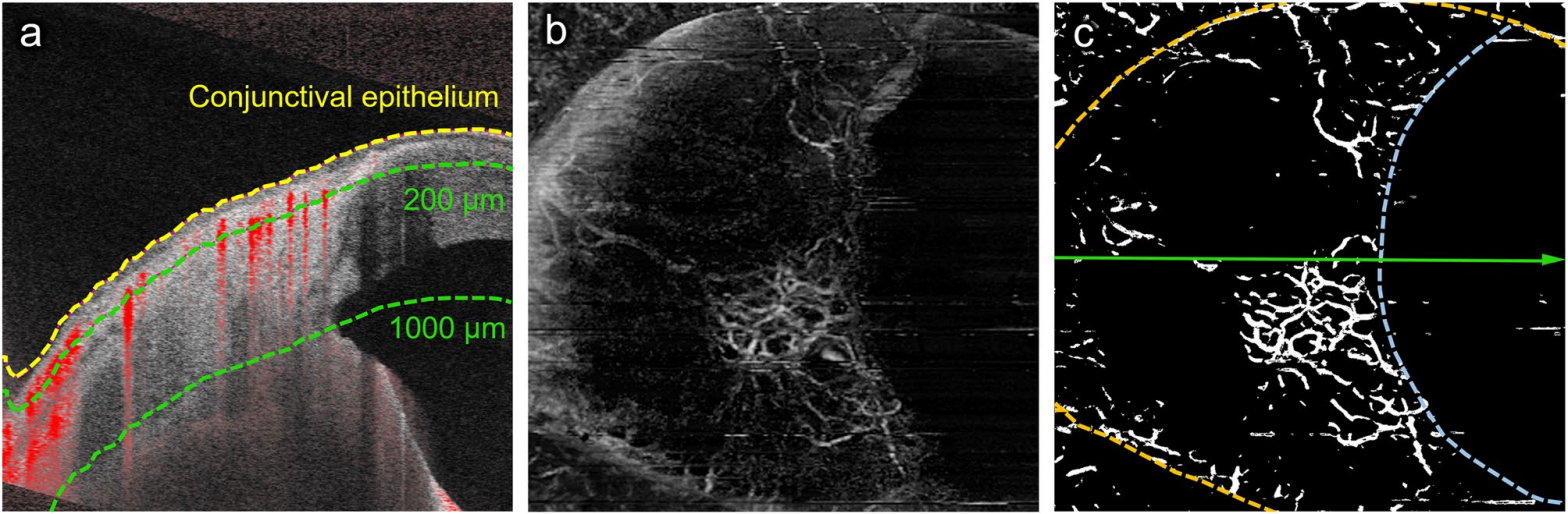
Measurements of vessel density using anterior segment (AS)-optical coherence tomography angiography (OCTA). (**a**) A cross-sectional AS-OCTA image overlying the B-scan image acquired at the green line in c. The yellow dotted line is the conjunctival epithelium, and the green dotted lines are at depths of 200 and 1000 µm from the conjunctival epithelium. The superficial (from the conjunctival epithelium to a depth of 200 µm) and deep (from a depth of 200 µm to 1000 µm from the conjunctival epithelium) layers are mainly composed of the conjunctiva and sclera, respectively. (**b**) A deep-layer AS-OCTA image on the nasal side around the corneal limbus of a left eye. (**c**) A binarized image of B. The aqua dotted line is the corneal marginal line, and the orange dotted lines are the eyelid margins.

The AS-OCTA examinations were performed using a swept-source optical coherence tomography (OCT; PLEX Elite 9000) system with a 10-dioptre optical adaptor lens developed by Carl Zeiss Meditec as previously reported^16,17,33,34^. A 3 × 3-mm scan was used to acquire the AS-OCTA images, which comprised 300 A-scans per B-scan repeated four times at each of the 300 B-scan positions. The AS-OCTA images of the nasal sclera and conjunctiva were acquired around the limbus for the analysis because the TM was incised/removed from the nasal quadrant during MIGS in this study for all cases. En face images of the superficial and deep layers were generated using built-in software (version 1.6; Carl Zeiss Meditec). The superficial layer (from the conjunctival epithelium to a depth of 200 μm) mainly comprised the conjunctiva, and the deep layer (from a depth of 200 μm to a depth of 1000 μm) mainly comprised the sclera (Fig. 2a). Projection artifacts caused by superficial images were removed from the deep images using the projection artifact removal algorithm in the built-in software. Because ripasudil was reported to increase deep VD as a short-term effect, ripasudil instillation was stopped at least 1 week before surgery and the cessation of ripasudil was maintained after surgery when applicable.

### Measurement of AS-OCTA parameters

The VD was measured using an en face image of the superficial and deep layers in the nasal region. Vessel binarization was performed using the Trainable Weka Segmentation plugin for Fiji (free downloadable software, https://fiji.sc) to suppress the effects of noise in the AS-OCTA images^32,35–37^. First, we converted the original images to an eight-bit grayscale image using Fiji. A representative OCTA image was used to train the software to differentiate vessels from the background by drawing representative lines inside and outside the selected vessels. After confirming the adequacy of the vessel segmentation in the entire area of the image, the classifier was applied to all other images. Based on the binarized image, the VD was shown as a ratio of pixels representing vessels of the entire area. The superficial and deep VDs based on the AS-OCTA images were successfully calculated using this binarization algorithm in a previous study^32^. When the cornea and eyelid were included in the AS-OCTA image, they were carefully excluded from the regions of interest (Fig. 2b, c). AS-OCTA images before and 3 months after surgery were used for the analyses.

### Definition of surgical success

We defined primary surgical success as a postoperative IOP of ≤ 18 mmHg and an IOP reduction of ≥ 20% from the preoperative value with the same or lesser number of medications during the first postoperative year. The IOP values were evaluated at 3, 6, 9, and 12 months postoperatively. Treatment was considered a failure if the success criteria were not met during two consecutive visits after the third postoperative month. Surgical failure also included subsequent glaucoma surgery and loss of light perception. The percent change in IOP was evaluated at 12 months postoperatively.

### Surgical procedure

Patients underwent MIGS within the area including the nasal hemisphere of the eye with or without phacoemulsification and intraocular lens implantation. The choice of MIGS, such as trabectome surgery, microhook trabeculotomy, and suture trabeculotomy, was made by the surgeon; however, TM in the nasal quadrant was incised or removed for all cases. Trabectome surgery was performed through a 1.70-mm temporal corneal incision, and the TM and the inner wall of the SC were removed nasally to form an arc of 100°–120°^5,6^. Microhook trabeculotomy was performed through a temporal corneal port, and the TM and the inner wall of the SC was incised nasally using a straight hook (Tanito ab interno Trabeculotomy Micro-hook; Inami & Co., Ltd., Tokyo, Japan) to create a 100°–120° arc^7^. During suture trabeculotomy, 5-0 nylon was inserted in the SC and pulled out from the location where the nylon suture stopped after the TM, and the inner wall of the SC at the nasal area were incised using a Tanito Micro-hook through a temporal corneal port, resulting in the creation of a 180°–360° arc^8,9^. When the patients had a visually significant cataract requiring surgical treatment, phacoemulsification and intraocular lens implantation were simultaneously performed through a clear corneal incision.

After the surgical procedure, three types of drops, 0.5% moxifloxacin, 0.1% betamethasone sodium phosphate, and 2% pilocarpine hydrochloride, were administered four times per day. For patients who underwent cataract surgery, bromfenac sodium hydrate was also prescribed. Betamethasone sodium phosphate was changed to 0.1% fluorometholone a few days after surgery. They were all discontinued within 3 months postoperatively. Anti-glaucoma eye drops were discontinued immediately after the surgery and were resumed when the postoperative IOP exceeded the target IOP for each case.

### Statistical analyses

The categorical data are presented as numbers and percentages. The continuous data are reported as mean ± standard deviation if they are normally distributed. The normality of the data was assessed using the Shapiro–Wilk test and visual inspection of histograms. Inter-group comparisons of categorical variables were conducted using Fisher’s exact test, and inter-group comparisons of continuous variables were conducted using an unpaired *t*-test for normally distributed data or the Mann–Whitney *U* test for nonparametric data. The Wilcoxon signed-rank test was used to analyze the difference in VD before and after MIGS. Univariate and stepwise multiple linear regression analyses were performed to identify factors affecting the change in deep VDs following surgery. Power calculations for statistical analyses were performed using G*Power software (version 3.1.9.6; Heinrich-Heine-Universität Düsseldorf, Düsseldorf, Germany). All statistical analyses were performed using SPSS version 22.0 for Windows (IBM Japan, Tokyo, Japan). *P* < 0.05 was considered statistically significant.

## Supplementary Information

Supplementary Information 1.

Supplementary Information 2.

## Data Availability

The data that support the findings of this study are available from the corresponding author, TA, upon reasonable request.
