# Supplementary material for: Changes in the deep vasculature assessed using anterior segment OCT angiography following trabecular meshwork targeted minimally invasive glaucoma surgery
Source: Sci Rep. 2022 Oct 13;12:17187. doi: 10.1038/s41598-022-22104-4 (PMC9561613; doi:10.1038/s41598-022-22104-4)
Supplement: Supplementary file 1 — Supplementary Information 1. [file 41598_2022_22104_MOESM1_ESM.pdf]

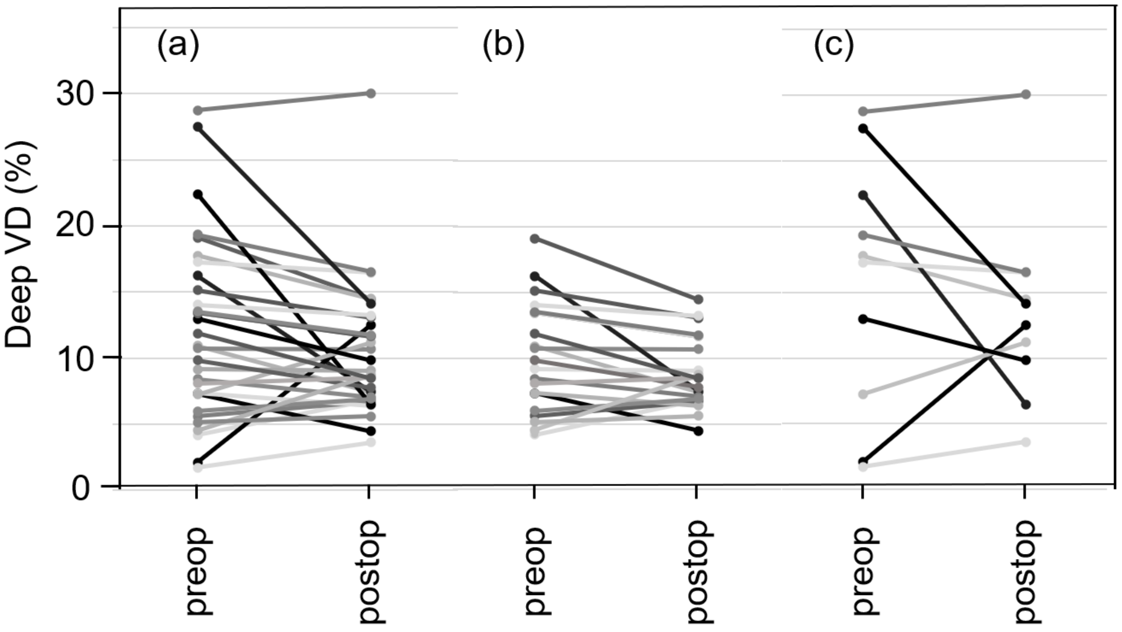

Supplementary Figure 1. Change in deep vessel density (VD) before and after minimally invasive glaucoma surgery. Each line graph shows the individual change in the deep VD before and after minimally invasive glaucoma surgery. **a.** Changes in the deep VDs for all cases (N = 31). **b.** Changes in the deep VDs in the successful group (N = 21). **c.** Changes in the deep VDs in the unsuccessful group (N = 10).
